# Optimization of non-denaturing protein extraction conditions for plant PPR proteins

**DOI:** 10.1371/journal.pone.0187753

**Published:** 2017-11-07

**Authors:** Nuria Andrés-Colás, Dominique Van Der Straeten

**Affiliations:** Laboratory of Functional Plant Biology, Department of Biology, Ghent University, Ghent, Belgium; Instituto de Biologia Molecular y Celular de Plantas, SPAIN

## Abstract

Pentatricopeptide repeat proteins are one of the major protein families in flowering plants, containing around 450 members. They participate in RNA editing and are related to plant growth, development and reproduction, as well as to responses to ABA and abiotic stresses. Their characteristics have been described *in silico*; however, relatively little is known about their biochemical properties. Different types of PPR proteins, with different tasks in RNA editing, have been suggested to interact in an editosome to complete RNA editing. Other non-PPR editing factors, such as the multiple organellar RNA editing factors and the organelle RNA recognition motif-containing protein family, for example, have also been described in plants. However, while evidence on protein interactions between non-PPR RNA editing proteins is accumulating, very few PPR protein interactions have been reported; possibly due to their high instability. In this manuscript, we aimed to optimize the conditions for non-denaturing protein extraction of PPR proteins allowing *in vivo* protein analyses, such as interaction assays by co-immunoprecipitation. The unusually high protein degradation rate, the aggregation properties and the high pI, as well as the ATP-dependence of some PPR proteins, are key aspects to be considered when extracting PPR proteins in a non-denatured state. During extraction of PPR proteins, the use of proteasome and phosphatase inhibitors is critical. The use of the ATP-cofactor reduces considerably the degradation of PPR proteins. A short centrifugation step to discard cell debris is essential to avoid PPR precipitation; while in some cases, addition of a reductant is needed, probably caused by the pI/pH context. This work provides an easy and rapid optimized non-denaturing total protein extraction protocol from plant tissue, suitable for polypeptides of the PPR family.

## Introduction

Pentatricopeptide repeat containing proteins (PPRs) are found in some prokaryotes and almost all eukaryotes. In plants, they represent one of the largest protein families [1]. They play a major role in RNA metabolism [2, 3]. PPR proteins are essential in plant reproduction, where their absence often causes lethality [4–7]. In addition, they have been related to plant growth and development, through regulation of energy metabolism and responses to ABA, as well as to abiotic stresses [1, 8]. PPR proteins are also associated with photosynthetic defects, aberrant leaf development, changes in leaf pigmentation, tolerance to inhibitors of different biosynthetic pathways, and restoration of pollen fertility [1]. Most of the plant PPR proteins are located in mitochondria (65%) or chloroplasts (17%) [9]. PPR proteins are named based on the presence of around 35-amino-acid motifs, repeated in tandem [1]. Depending on the extra motifs, the PPR proteins are classified in different subfamilies (P-type and PLS) and subgroups (PLS, E, E+ and DYW). Each type of PPR protein is suggested to play a different task in RNA editing and to interact in modular editosomes for a complete editing event [10–12]. Besides *in silico* prediction of their characteristics [13], relatively little is known about their biochemical properties. Therefore, the working mechanisms of PPR proteins are by far not completely understood.

The characterization of native protein interactions is a crucial step to decipher the molecular mechanisms of biological processes. The identification of the complete set of interacting partners in multicomponent protein complexes, such as the PPR editosomes, is particularly difficult and requires the consideration of a broad range of protein properties. In contrast to the increasing number of reports on protein interactions between non-PPR RNA editing proteins [11], only few examples of PPR protein interactions have been reported. Proof of protein interaction among PPR proteins is mainly limited to yeast-two-hybrid or *in vitro* pull-down assays [14–20]. Those interaction assays, performed out of the natural *in vivo* protein context, may lead to false positives, given an inappropriate spatio-temporal expression (cell type, subcellular compartment, lifecycle-time…), or even to false negatives, given the lack of unknown partners or intermediate processes. Only three papers reported *in vivo* interactions of PPR proteins in *Arabidopsis thaliana*: a first one showed bimolecular fluorescence complementation (BiFC) in *A*. *thaliana* seedlings transiently transformed with two PPR proteins (CRR4 and DYW1) which interact in chloroplasts [21]; another study revealed interaction of the PPR protein/mitochondrial editing factor 13 (MEF13) with MORF3 and MORF8 in mitochondria, also by BiFC [15]; and a last one presented immunoprecipitation data from stably transformed *A*. *thaliana* plants, showing interaction of the PPR protein Required for ACC RNA Editing 1 (RARE1) with RIP1 in chloroplasts [14]. Possible reasons for this relative delay in PPR protein research may be related to their biochemical characteristics such as high instability, unfolding properties and insolubility [22].

The most critical steps in any proteomic study are the protein extraction and the sample preparation. In general, outside their proper environment, which can vary considerably among cell compartments, proteins may misfold, aggregate and precipitate. Proteins are unstable when extracted from their *in vivo* context, especially in plants [23]. Depending on the particular biochemical properties of each protein, the factors and conditions which ensure protein stability upon extraction can vary considerably. Moreover, these conditions must be compatible with the downstream protein analysis. PPR proteins are known for their low expression levels and for being notoriously difficult to express, enrich and purify. In this sense, optimization studies to facilitate and improve PPR protein extraction approaches are meaningful. The fact that plant PPR proteins were found to be part of complexes with other proteins or RNA, sometimes attached to membranes [24], could also complicate the extraction of proteins in a non-denaturing state, which would maintain *in vivo* protein interactions. Difficulties in obtaining soluble PPR proteins upon expression in heterologous systems, such as *Escherichia coli* or yeast, have been repeatedly reported [22, 25–27]. The specific isoelectric point (pI) of a protein is also crucial for its solubility. PPR proteins can have a great variability of pI, such as the Etype PPRs with a pI ranging from 5.23 to 9.11 [28]. Moreover, the phosphorylation of some non-PPR editing factors was described as a crucial step for their proper subcellular localization [29]. Given that many PPR proteins have an ambiguous localization, a similar phosphorylation mechanism could be involved in determining their subcellular localization. In contrast, the phosphorylation of other proteins provides a hallmark for degradation in the ATP-dependent ubiquitin/proteasome pathway [30]. Furthermore, ATP and divalent cations, such as zinc, have been described as necessary cofactors for the RNA editing activity [31, 32]. Therefore, the common use of EDTA to inhibit the activity of metalloproteases could interfere with the functional state of the editing factors.

Tissue disruption, inhibition of secondary metabolites, and solubilization of proteins without affecting possible protein interactions is the goal of this work. In this manuscript, we report on the difficulties faced when working with certain PPR proteins. We demonstrate the influence of different conditions and components in the protein extraction buffer. We aimed to provide a straightforward protocol for non-denaturing PPR protein extraction from transiently transformed *Nicotiana benthamiana* leaves for protein blotting detection. Allowing simple and effortless analysis, this optimized procedure could help the scientific community when working with PPR or similarly problematic proteins, thus helping to increase the knowledge about one of the major protein families in flowering plants.

## Results

### Protein degradation over time

**Fig 1:**
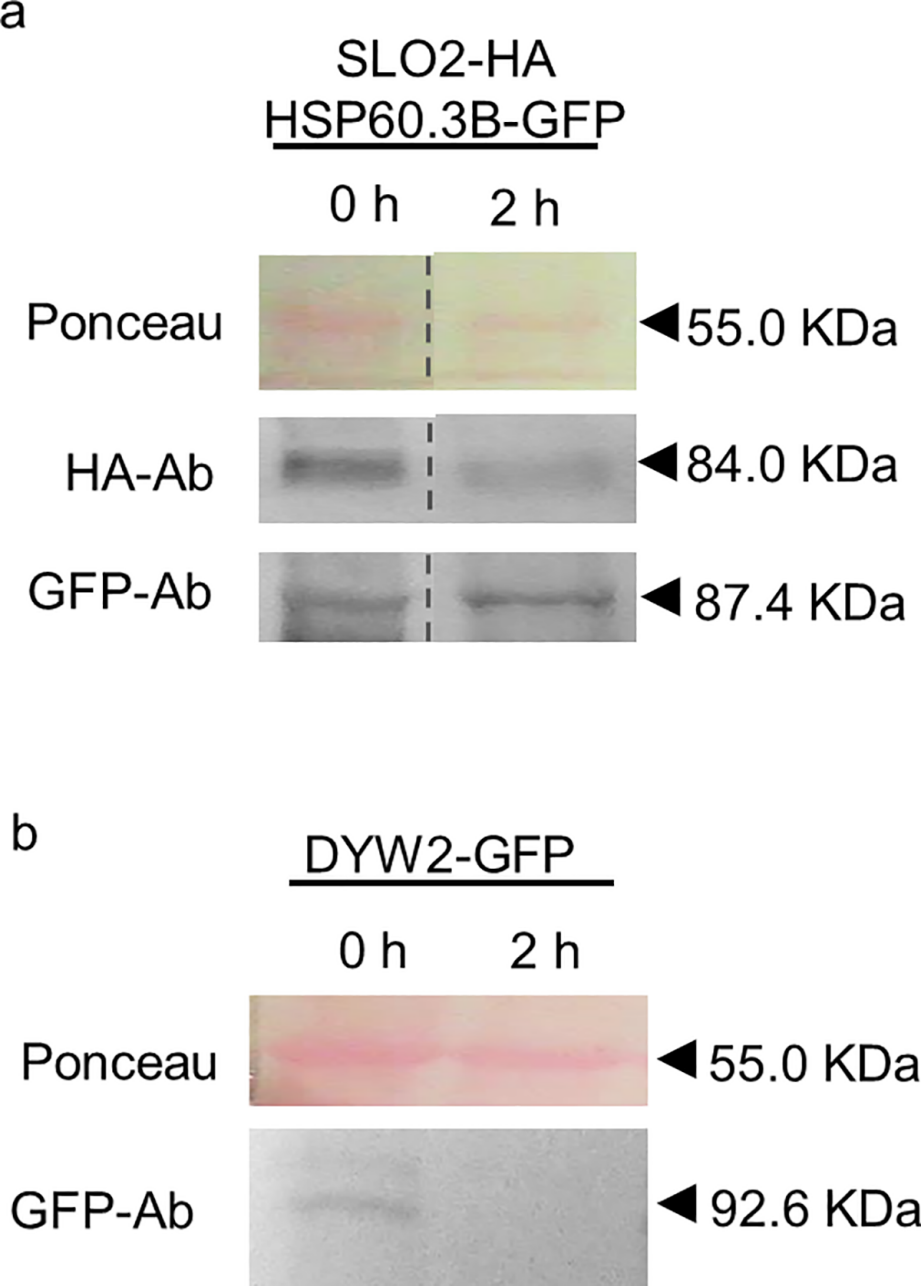
Degradation of PPR proteins after extraction. Total protein extract from *N*. *benthamiana* leaves infiltrated with SLO2-HA construct together with HSP60.3B-GFP **(a)** or DYW2-GFP alone **(b)**. Aliquots at 0 and 2 h after extraction were analysed by western blot, stained with Ponceau and probed with anti-HA and anti-GFP antibodies (HA-Ab and GFP-Ab, respectively). The respective molecular weights are: SLO2-HA, 84.03 kDa; DYW2-GFP, 92.56 kDa; and HSP60.3B-GFP, 87.42 kDa. The Ponceau membrane staining of the most intense band at 55 kDa (presumably Rubisco) was used as a loading control. Full-length blots are shown in S1 Fig. Histograms of GFP/HA-tagged protein, relative to Ponceau, are shown in S2 Fig.

Most of the *in vivo* protein analyses, such as, for instance, co-immunoprecipitation experiments, require a relative lengthy protocol. A first hurdle when working with PPR proteins was the unusually low protein level leading to failure of protein detection at the end of the experiment. This problem is not faced when working with most other proteins. To check whether this absence of protein detection was due to an inefficient protein extraction or to protein degradation after the extraction step, we decided to analyse the protein levels at time 0 and 2 h after protein extraction, by blotting. With this purpose, two different types of PPR proteins, the mitochondrial E+-type PPR protein SLO2 (At2g13600) [8] and the DYW-type PPR protein DYW2 (At2g15690), dual localized in mitochondria and chloroplasts [9, 33], were tagged with the human influenza hemagglutinin (HA) epitope or the green fluorescent protein (GFP) protein. Constructs of the corresponding tagged proteins were transiently expressed in *N*. *benthamiana* leaves. The mitochondrial chaperone protein HSP60.3B (At3g23990) was also used as a non-PPR protein to compare with, and was co-infiltrated together with the SLO2 construct to allow comparative analysis. After 3 days of infiltration, proteins were extracted from the leaves with a standard non-denaturing protein extraction buffer. We chose an extraction buffer slightly modified from the one used previously for chloroplastic PPR immunoprecipitation from stably transformed *A*. *thaliana* plants [14] and the one for weak protein-protein interactions indicated in the μMACS Epitope Tag Protein Isolation Kit protocol (Miltenyi Biotec) (see Methods section for the exact buffer composition); 2x EDTA-free protease inhibitor cocktail was added according to the manufacturer’s recommendations against very high proteolytic activity. Each sample was split in two: one half was immediately incubated with sample buffer at 65°C and subsequently transferred to 4°C (0 h sample); the other half was kept on ice for 2 h before incubation at 65°C with the sample buffer (2 h sample). Equal volumes of both halves were loaded onto a polyacrylamide gel and analysed by blotting. Ponceau staining was used to check equal loading. All samples were analysed directly after preparation to avoid protein loss by storage and/or freezing steps. As shown in Fig 1, the PPR proteins were detected at time 0 h after extraction but the protein levels were considerably reduced after 2 h on ice. This effect was not observed for the chaperone protein, for which the level was unaltered between 0 and 2 h. These results illustrate a protein degradation problem over time, specific for PPR proteins. Furthermore, it gave an incentive to eliminate the standard 5-30 minutes extraction incubation step, commonly found in protein extraction protocols.

### Avoiding protein degradation during extraction

**Fig 2:**
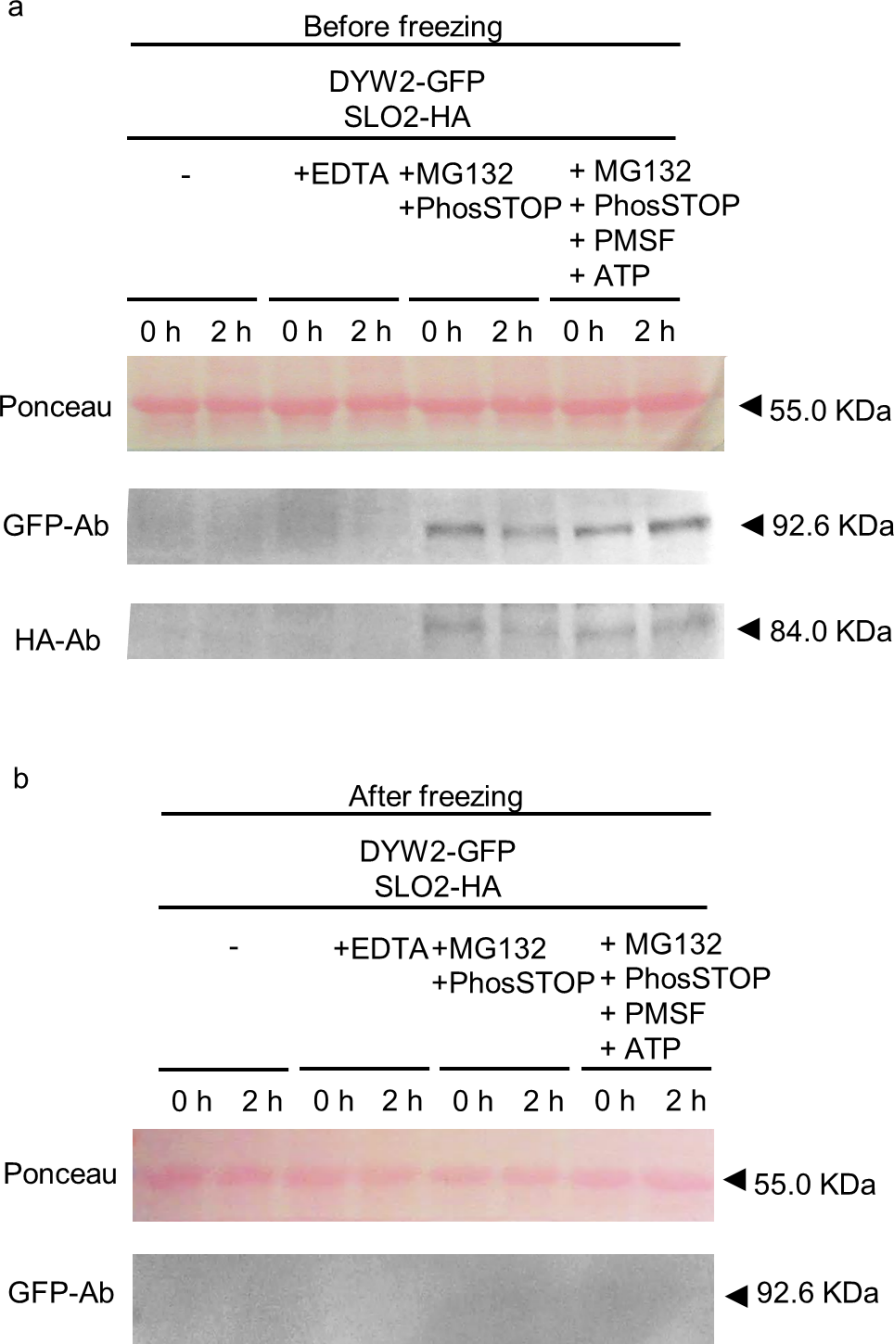
Optimization of the extraction buffer components for PPR proteins. Total protein extracts from *N*. *benthamiana* leaves co-infiltrated with SLO2-HA and DYW2-GFP constructs. The extraction buffer used was complemented with 2 mM EDTA, 50 μM MG132 proteasome inhibitor, 1x phosphatase inhibitor, 5 mM ATP and/or 1 mM PMSF. Aliquots at 0 and 2 h after extraction, and before **(a)** or after **(b)** o/n freezing at -20°C, were analyzed by western blot, stained with Ponceau and probed with anti-HA and anti-GFP antibodies (HA-Ab and GFP-Ab, respectively). The respective molecular weights are: SLO2-HA, 84.03 kDa; and DYW2-GFP, 92.56 kDa. The Ponceau membrane staining of the most intense band at 55 kDa (presumably Rubisco) was used as a loading control. Full-length blots are shown in S1 Fig. Histograms of GFP/HA-tagged protein, relative to Ponceau, are shown in S2 Fig.

Given the observed high instability of PPR proteins, we decided to test the effect of different components of the protein extraction buffer on inhibition of their particularly high protein degradation rate. For this purpose, tagged versions of the SLO2 and DYW2 PPR proteins were co-infiltrated in *N*. *benthamiana* leaves. After 3 days of infiltration, equal amounts of grinded material from the same leaf were taken and extracted simultaneously with the different protein extraction buffers. Each extracted sample was also split in two to compare time 0 and 2 h after protein extraction. Each sample was immediately incubated at 65°C with sample buffer after the corresponding time. Equal volumes of each sample were loaded onto a polyacrylamide gel and analysed by blotting. Ponceau staining was used to assess equal loading. Our starting point was the same standard non-denaturing protein extraction buffer used before, containing EDTA-free protease inhibitor cocktail. First, we tested the effect of EDTA as an inhibitor of metalloproteases in the extraction buffer. An EDTA concentration ranging from 1 to 5 mM was previously used in protocols for protein extraction from *N*. *benthamiana* leaves, and subsequent immunoprecipation assays [34, 35]. We decided to test an intermediate concentration of 2 mM EDTA. As shown in Fig 2, no significant differences were observed when comparing the standard non-denaturing protein extraction buffer with or without EDTA.

Second, we tested the effect of a phosphatase inhibitor. Some editing factors were described to be phosphorylated to allow proper subcellular localization, while their dephosphorylation could lead to a non-functional protein susceptible to degradation by the 26S proteasome. Therefore, we decided to test a proteasome inhibitor together with a phosphatase inhibitor. The phosphatase inhibitor was used at the concentration recommended by the manufacturer, and the concentration of the proteasome inhibitor was chosen according to the literature for protein extraction or immunoprecipitation purposes [36, 37]. As shown in Fig 2A, the addition of phosphatase inhibitor together with proteasome inhibitor increased considerably the amount of protein detected after extraction, for both SLO2 and DYW2 PPR proteins.

Third, we tested the effect of adding ATP and extra protease inhibitor. Some editing factors were described to require ATP as a cofactor for the RNA editing activity; consequently, it is possible that, over time, the lack of this cofactor could lead to degradation of the non-functional editing factors. We chose an ATP concentration of 5 mM, considering the published ATP requirements for editing activity [31]. Additional protease inhibitor (phenylmethylsulfonyl fluoride, PMSF) at a 1 mM concentration [35, 38] was also used. As shown in Fig 2A, the addition of ATP together with PMSF decreased PPR protein degradation after 2 h of extraction, as compared with extraction buffers lacking them.

Furthermore, by analysing the same samples after an overnight storage at -20°C, we corroborated that the freezing storage of the extracted proteins results in PPR protein losses (Fig 2).

### Preventing protein precipitation

**Fig 3:**
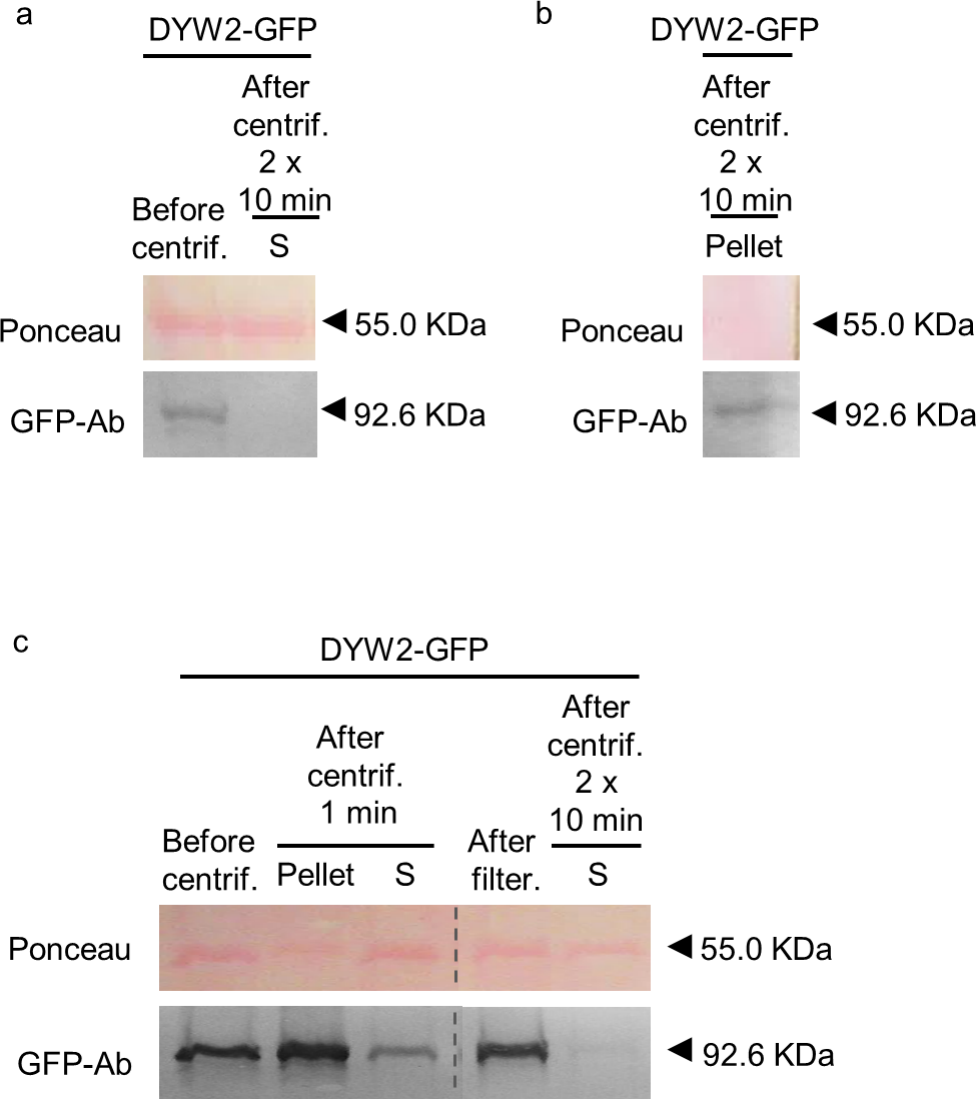
Optimization of the removal of cell debris during the extraction of PPR proteins. Total protein extract from *N*. *benthamiana* leaves infiltrated with DYW2-GFP construct. The extraction buffer used was complemented with 50 μM MG132 proteasome inhibitor, 1x phosphatase inhibitor, 5 mM ATP and 1 mM PMSF. Total protein extracts were centrifuged for 1 min or twice for 10 min at 12000 rpm and 4°C or filtered on a 40 μM nylon mesh. Samples before and after centrifugation or filtering, and the pellet, were analyzed by western blot, stained with Ponceau and probed with anti-GFP antibodies (GFP-Ab). S, supernatant. Centrif., centrifugation. Filter., Filtering. The respective molecular weight is: DYW2-GFP, 92.56 kDa. The Ponceau membrane staining of the most intense band at 55 kDa (presumably Rubisco) was used as a loading control. Full-length blots are shown in S1 Fig. Histograms of GFP/HA-tagged protein, relative to Ponceau, are shown in S2 Fig.

Protein extraction protocols contain a centrifugation step to discard the cell debris. For *in vivo* protein analyses such as co-immunoprecipitation assays, this centrifugation step is usually much longer (10–30 minutes). After centrifugation, a white pellet, different from the green pellet containing cell debris, was obtained when working with PPR proteins. To check whether the DYW2 PPR protein was precipitating during the centrifugation step, we decided to analyse the protein level in aliquots taken before and after the centrifugation step. Each sample taken was immediately incubated at 65°C with sample buffer. Equal volumes of the samples were loaded onto a polyacrylamide gel and analysed by blotting. Ponceau staining was used to assess equal loading. As shown in Fig 3A, the protein was detected before the centrifugation but not in the supernatant after centrifugation. Therefore, we decided to analyse the resulting pellet from the same experiment to exclude a degradation issue. The pellet was taken and homogenized in sample buffer, incubated at 65°C, and loaded onto a polyacrylamide gel. As shown in Fig 3B, the protein was detected after centrifugation in the pellet, indicating protein precipitation.

Subsequently, we decided to check shorter centrifugation steps, and to test other methods to discard cell debris from the tissue extract, such as filtering. With this purpose, an extracted sample was split in three: two halves were centrifuged and the other half filtered. Aliquots were taken before splitting the sample, after centrifugation, and after filtering. Each sample taken was immediately incubated at 65°C with sample buffer. Equal sample volumes were loaded onto a polyacrylamide gel and analysed by blotting. Ponceau staining was used as a loading control. The pellet was also taken and homogenized in sample buffer, incubated at 65°C and loaded onto the same gel. As shown in Fig 3C, reducing the centrifugation step to 1 min was sufficient to recover a detectable amount of PPR protein while avoiding excessive precipitation. A centrifugation of 2 minutes was already too much and resulted in precipitation of all the protein (data not shown). Moreover, a filtering step with a 40 μM nylon mesh was also effective in retaining a considerable amount of detectable protein (Fig 3C).

**Fig 4:**
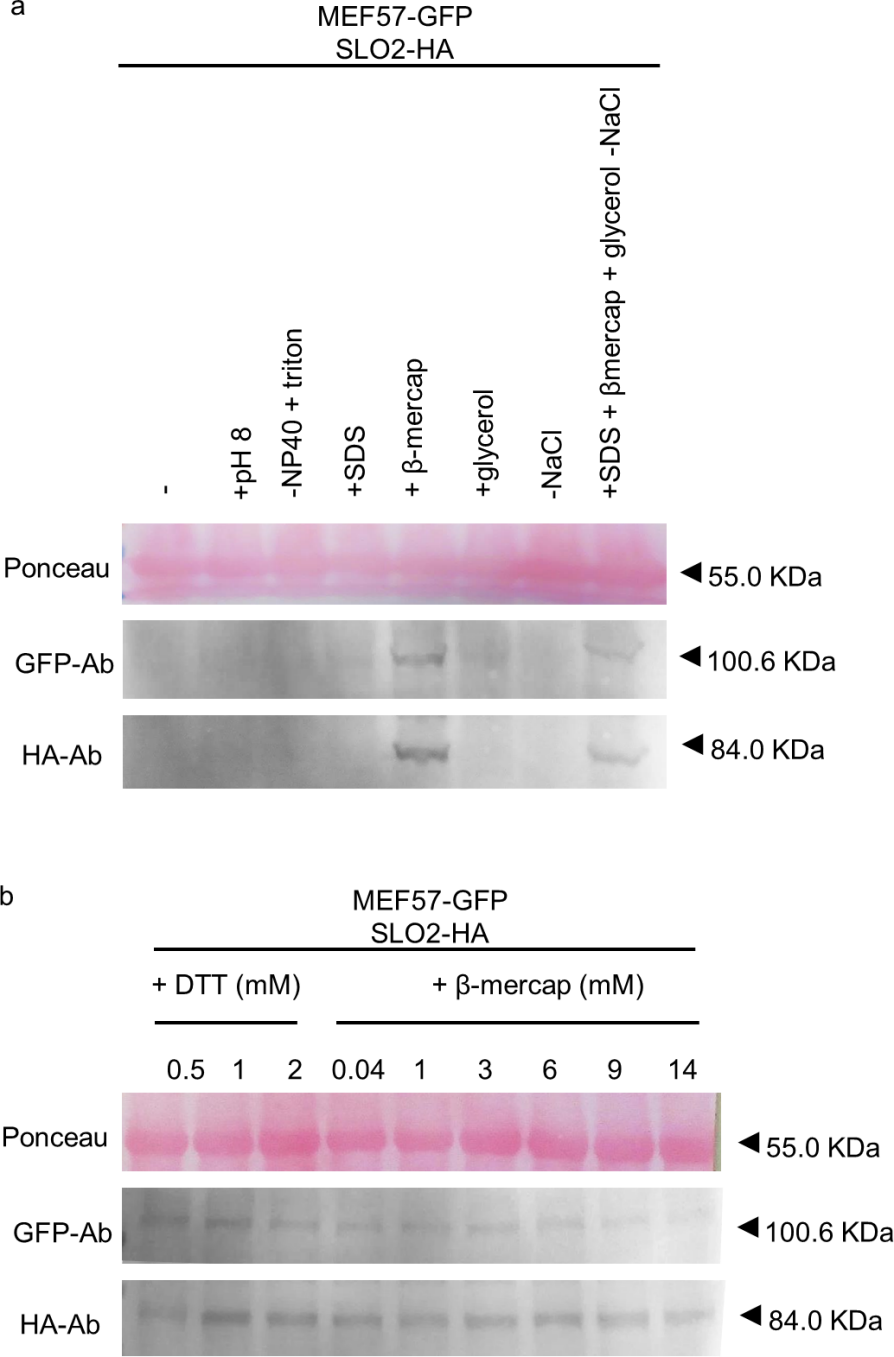
Optimization of the extraction buffer components for MEF57. Total protein extracts from *N*. *benthamiana* leaves infiltrated with SLO2-HA construct together with MEF57-GFP. The extraction buffer was complemented with 50 μM MG132 proteasome inhibitor, 1x phosphatase inhibitor, 5 mM ATP and 1 mM PMSF. The pH, the NP40 detergent and the NaCl content were modified to pH 8, 1% Triton X-100 and 0 mM NaCl **(a)**. 0.1% SDS, 14 mM β-mercaptoethanol and 10% glycerol were tested **(a)**. Several concentrations of DTT and β-mercaptoethanol were also tested **(b)**. Samples were analyzed by western blot, stained with Ponceau and probed with anti-HA and anti-GFP antibodies (HA-Ab and GFP-Ab, respectively). The respective molecular weights are: SLO2-HA, 84.03 kDa; DYW2-GFP, 92.56 kDa; MEF57-GFP, 100.62 kDa; and HSP60.3B-GFP, 87.42 kDa. The Ponceau membrane staining of the most intense band at 55 kDa (presumably Rubisco) was used as a loading control. Full-length blots are shown in S1 Fig. Histograms of GFP/HA-tagged protein, relative to Ponceau, are shown in S2 Fig.

We noticed that some of the PPR proteins, such as DYW-type PPR proteins DYW2 and MEF57 (At5g44230), are characterized by a high pI (7.51 and 7.78, respectively), close to the pH of the extraction buffer, which is at 7.5 pH units. This may cause their precipitation during the protein extraction procedure. Therefore, we decided to test other parameters of the extraction buffer that could affect the solubility of the proteins, especially in the case of the MEF57 protein, for which the previous modifications of the protocol were not sufficient to obtain an acceptable amount of protein. For this purpose, tagged versions of the SLO2 and MEF57 PPR proteins were co-infiltrated in *N*. *benthamiana* leaves. After 3 days of infiltration, equal amounts of grinded material from the same leaf were taken and extracted simultaneously with the different protein extraction buffers. Each sample was immediately incubated at 65°C with sample buffer. Equal volumes of each sample were loaded onto a polyacrylamide gel and analysed by blotting. Ponceau staining was used as a loading control. Our starting point was the standard non-denaturing protein extraction buffer used before, containing EDTA-free protease inhibitor cocktail, plus phosphatase and proteasome inhibitors, ATP, and extra PMSF protease inhibitor. First, we tried to slightly increase the pH of the extraction buffer above the pI of MEF57, to pH 8, but it did not improve protein detection (Fig 4A). Second, we tested the effect of reductants that could prevent insoluble protein conformations. The sodium dodecyl sulfate (SDS) reductant was tested at a 0.1% concentration, as indicated in the immunoprecipitation protocol of the μMACS Epitope Tag Protein Isolation Kit (Miltenyi Biotec). In addition, we used 14 mM β-mercaptoethanol, close to the highest concentration used in co-immunoprecipitation protocols in the literature [34, 39, 40]. As shown in Fig 4A, the addition of β-mercaptoethanol, but not SDS, was effective at extracting the SLO2 and MEF57 proteins. We also tested whether the addition of glycerol or the elimination of NaCl, helped to increase the protein level detected. A concentration of 10% glycerol was taken from the immunoprecipitation protocol of Abcam (www.abcam.com/technical) or as mentioned in the literature [41]. No significant changes were observed with respect to the standard extraction buffer without glycerol or with NaCl (Fig 4A). Furthermore, we tested whether stronger detergents such as Triton X-100 (slightly more hydrophilic than NP40) could help in extraction of these particular PPR proteins. Triton X-100 was used at 1%, as the highest concentration advised in literature [34, 41] and applied in the immunoprecipitation protocol of Miltenyi (μMACS Epitope Tag Protein Isolation Kit protocol, Miltenyi Biotec). No improvement was observed, with respect to the standard extraction buffer (Fig 4A).

Finally, we checked different concentrations of β-mercaptoethanol, trying to reduce the negative side effects of this component in protein analyses *in vivo*. β-mercaptoethanol was used in a concentration range of 0.04–14 mM, according to the co-immunoprecipitation protocols in the literature [34, 39, 40]. DL-Dithiothreitol (DTT) was tested as an alternative reductant. A concentration of 2 mM DTT was chosen from co-immunoprecipitation experiments of mitochondrial PPR proteins in different plants [24, 42], and two lower concentrations (1 and 0.5 mM) were also tested. From Fig 4B, we can conclude that the concentration of DTT and β-mercaptoethanol could be reduced to 1 and 0.04 mM, respectively, with no detrimental effect on the protein extraction efficiency.

**Fig 5:**
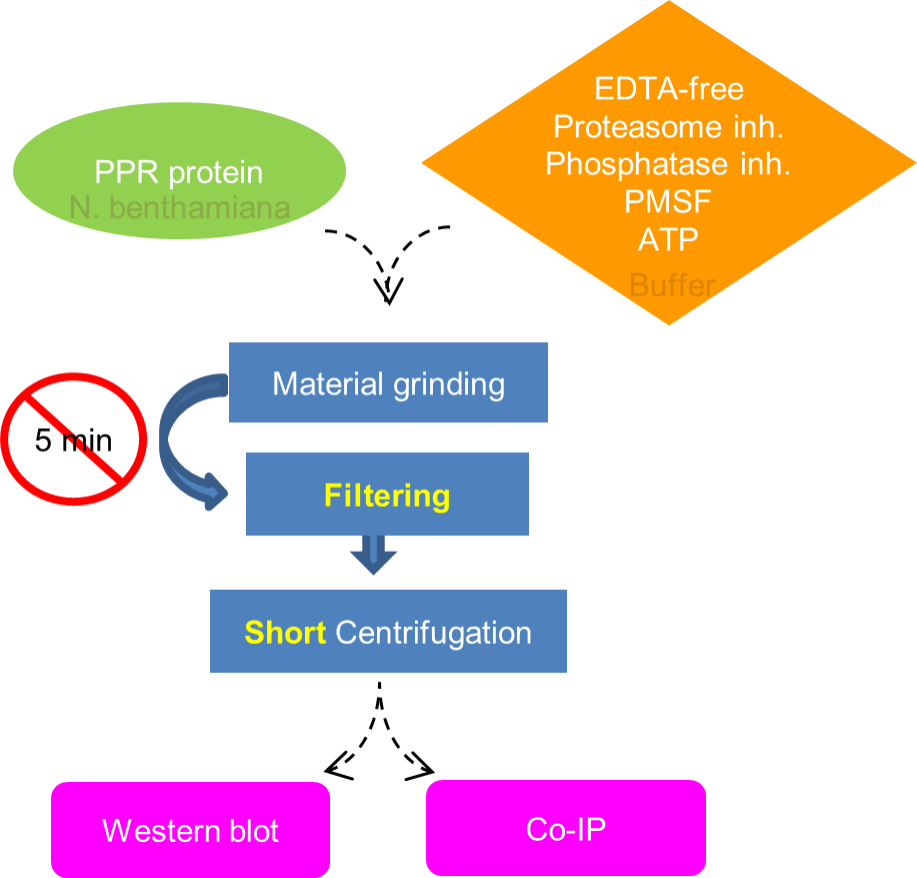
Flowchart of the optimized protocol.

A flowchart of the optimized protocol is shown in Fig 5. The established protocol was successful on downstream co-immunoprecipitation analysis [43], showing its applicability to *in vivo* analyses.

## Discussion

The standard protein extraction buffer used for PPR proteins in this paper was supplemented with an EDTA-free protease inhibitor cocktail, which lacks any specific inhibitor of metalloproteases. Given that some RNA editing factors have been described to require divalent cations, which are chelated by EDTA, the extraction buffer was kept free of EDTA to avoid the non-functionality of the PPR proteins that could possibly interfere with their solubility.

We emphasise the importance of working with freshly extracted proteins, since a freezing and thawing step leads to loss of detectable protein. Accordingly, we recommend designing the timing of the experiment such that western blotting can be performed immediately after the protein extraction. If storage is unavoidable, it is recommended to perform it at 4°C and as brief as possible.

Furthermore, when using a reductant to prevent protein precipitation during the extraction, it is important to subsequently diminish the concentration of the reducing agent. The reductant could interfere in successive analyses such as a co-immunoprecipitation assay, either affecting the protein-protein interaction or the antibody properties. Moreover, a delicate balance in the amount of reductant is needed, on one hand being high enough to prevent protein precipitation, on the other hand being low enough to avoid loss of protein–protein interaction or of antibody integrity. Such a balance could be quite tricky. Therefore, instead of using reductants to prevent protein precipitation, and since centrifugation could provoke aggregation and subsequent precipitation of the proteins, we propose to use a short centrifugation step to discard the cell debris.

For some protein analyses such as a co-immunoprecipitation assay, the absence of cell debris and aggregates is of utmost importance to avoid interferences that could result in a false positive. Given that both filtering and 1-minute-centrifugation methods, shown to be effective to extract PPR proteins, leave some cell debris in the sample, we recommend the combination of a filtering step followed by a 1-minute-centrifugation. In our hands, this combination proved to be efficient enough to retain PPR proteins while avoiding cell debris for successive co-immunoprecipitation assays [43].

## Conclusions

The high protein degradation rate, the aggregation properties, the need of ATP as a co-factor, and the high pI are key aspects to be considered when extracting PPR proteins in a non-denatured state. Therefore, the use of specific proteasome and phosphatase inhibitors is a critical point when extracting PPR proteins. ATP is also needed to avoid protein degradation once the PPR proteins have been extracted. Short centrifugation steps are another essential element to avoid PPR precipitation. Last but not least, in some cases, a reductant such as DTT or β-mercaptoethanol can be needed to prevent PPR protein precipitation, presumably caused by the pI/pH context.

The observed high degradation rate of PPR proteins could explain the low number of PPR protein interactions described *in vivo*. We provide an easy and rapid protocol for non-denaturing PPR protein extraction from transiently transformed *N*. *benthamiana* leaves, with suggestions for optimization for the proteins under study. The resulting protocol is useful for *in vivo* protein analyses such as co-immunoprecipitation experiments. We hope that this straightforward optimized protocol will facilitate research on this major protein family, or similarly problematic proteins in plants.

## Materials and methods

### Plant materials and growth conditions

*N*. *benthamiana* leaves were used. Plants were grown in a 16 h light/8 h dark (long day) photoperiod, under white fluorescence light (75 μM m^–2^ s^–1^), and at 22°C, for 2–3 months.

### Plasmid constructs

Plasmids were constructed as previously described [42]. Briefly, the complete coding sequences of interest were obtained from *A*. *thaliana* cDNA by HiFi PCR using attB-flanked primers (S1 Table), which introduced the adequate sites for the subsequent cloning by the Gateway system (Invitrogen) into the pDONR221 vector via BP reaction, and then confirmed by sequencing. The verified fragments were cloned under the control of the constitutive CaMV 35S promoter via LR reaction into the pK7WGF2 vector [44] for C-terminal fusion with the GFP; and in the pGWB514 vector (kindly provided by Tsuyoshi Nakagawa [45]) for C-terminal fusion with a 3xHA epitope.

### Protein expression and extraction

Proteins were expressed as previously described [42]. 2-day cultures of *Agrobacterium tumefaciens* harbouring the desired constructs and the silencing-suppressor p19 plasmid [46] were pelleted, diluted to OD_600_ 0.1–0.5 in infiltration buffer [0.5% (w/v) D-glucose; 10 mM MES; 10 mM MgCl_2_; 0.1 mM acetosyringone], and co-infiltrated in young *N*. *benthamiana* leaves. Samples were taken 3–6 days after infiltration.

Total proteins were extracted from plant material expressing the constructs of interest (transiently transformed *N*. *benthamiana* leaves). The initial extraction buffer used in this manuscript was slightly modified from the one used previously for chloroplastic PPR immunoprecipitation from stable transformed *A*. *thaliana* plants [14], and the one for weak protein-protein interactions indicated in the μMACS Epitope Tag Protein Isolation Kit protocol (Miltenyi Biotec), plus EDTA-free protease inhibitor cocktail according to the manufacturer’s recommendations for very high proteolytic activity: 50 mM Tris-HCl pH7.5, 150 mM NaCl, 1% NP40 (Igepal CA630), 2x protease inhibitor mix cocktail without EDTA (EDTA-free Complete, Roche REF04 693 132 001). As a result of this manuscript, for an optimized composition of the extraction buffer, suitable for PPR proteins, the initial extraction buffer was complemented with: 1x phosphatase inhibitor (PhosSTOP, Roche), 50 μM MG132 proteasome inhibitor (C2211, SIGMA), 5 mM ATP and 1 mM PMSF. SDS 0.1%, β-mercaptoethanol 14 mM,glycerol 10%, or varying concentrations of DTT and β-mercaptoethanol were also tested, as indicated in this manuscript. The pH, the NP40 detergent, and the content of NaCl were modified to pH 8, Triton X-100 1% and 0 mM NaCl, as indicated, for testing. As a result of this study, for optimized conditions of protein extraction suitable for PPR proteins, grinded samples were filtered with 40 μM nylon mesh and centrifuged for 1 min at 12000 rpm and 4°C, to discard the cell debris.

### Western blotting

Protein samples were incubated with 2x sample buffer [Tris-HCl 250 mM, pH 6.8; glycerol 20% (v/v); SDS 4% (p/v); β-mercaptoethanol 10% (v/v) and bromophenol blue 0.025% (p/v)] for 5 min at 65°C, immediately after extraction. 30 μl of the protein samples were run in an 8% SDS-PAGE gel and blotted on a 0.45 μm nitrocellulose membrane (Amersham Protran) at 4°C. The membrane was stained with 0.1% Ponceau S in 1% acetic acid for 5 min. The background stain was removed by treatment with 1% acetic acid during 15 min before imaging. The Ponceau S was completely removed with T-TBS buffer [Tris-HCl 20 mM, pH 7.5; NaCl 0.5 M; Tween-20 0.1%]. The membrane was probed as previously indicated [42], with 1:100 anti-HA-HRP (clone 3F10 Roche) or 1:100 anti-GFP-HRP (A10260 Invitrogen) in T-TBS, and developed by a colorimetric assay (0.5 mg/ml DAB in 0.1 M imidazole pH 7 with 0.1 μl/ml 30% H_2_O_2_ and 54 μl/ml 0.6% CoCl_2_) [47]. Both antibodies were checked consecutively on the same membrane with a washing step (T-TBS buffer for 30 min) in between. The PageRule pre-stained protein ladder was used as size marker of 170, 130, 100, 70 (red), 55, 40, 35, 25 and 15 kDa (Thermo Scientific CN26616). The respective molecular weights are: SLO2-HA, 84.03 kDa; DYW2-GFP, 92.56 kDa; MEF57-GFP, 100.62 kDa; and HSP60.3B-GFP, 87.42 kDa.*Image J* software was used to quantify the band intensity.

## Supporting information

S1 Fig Full-length blots. Protein ladder shown in the first run (170, 130, 100, 70 (red), 55, 40, 35, 25 and 15 kDa). The respective molecular weights were: SLO2-HA, 66.41 kDa; DYW2-GFP, 92.56 kDa; MEF57-GFP, 100.62 kDa; and HSP60.3B-GFP, 87.42 kDa.
(PDF)

S2 Fig GFP/HA-tagged protein amount relative to Ponceau. Histograms showing the GFP/HA-tagged protein amount relative to Ponceau, according to band intensity in the blots.
(PDF)

S1 Table Oligonucleotides used for cloning. (PDF)
