# Supplementary figures and images for: Optimization of non-denaturing protein extraction conditions for plant PPR proteins
Source: PLoS One. 2017 Nov 7;12(11):e0187753. doi: 10.1371/journal.pone.0187753 (PMC5675432; doi:10.1371/journal.pone.0187753)

**S1 Fig. Full-length blots.** Protein ladder shown in the first run (170, 130, 100, 70 (red), 55, 40, 35, 25 and 15 kDa).

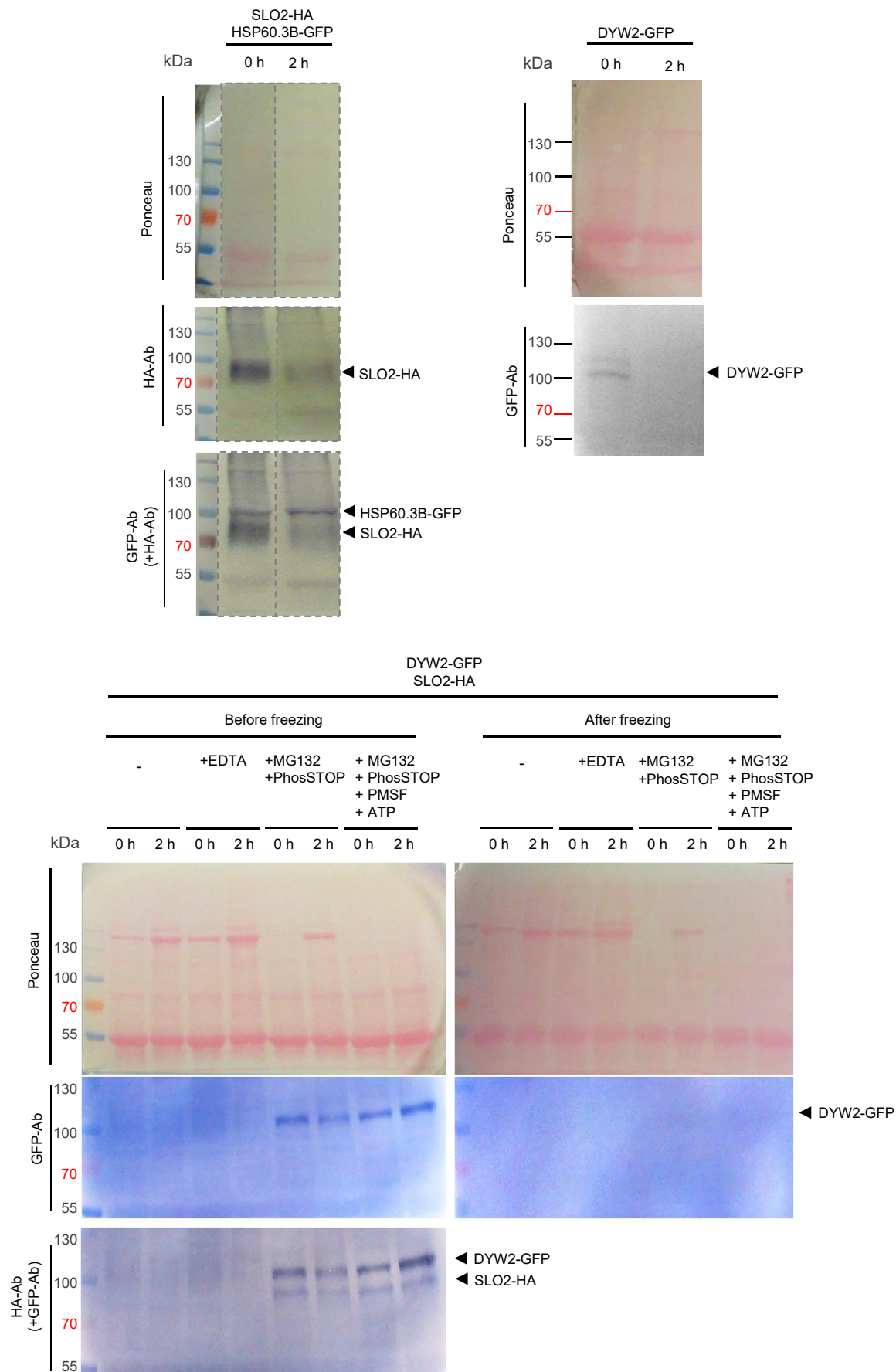

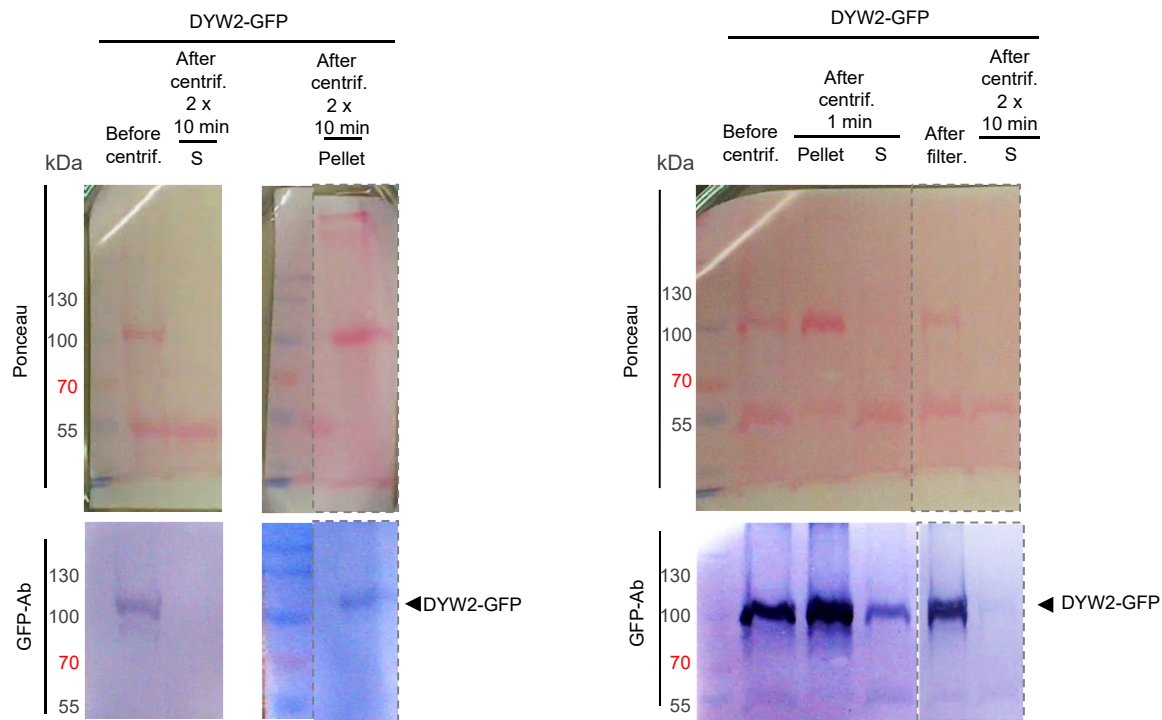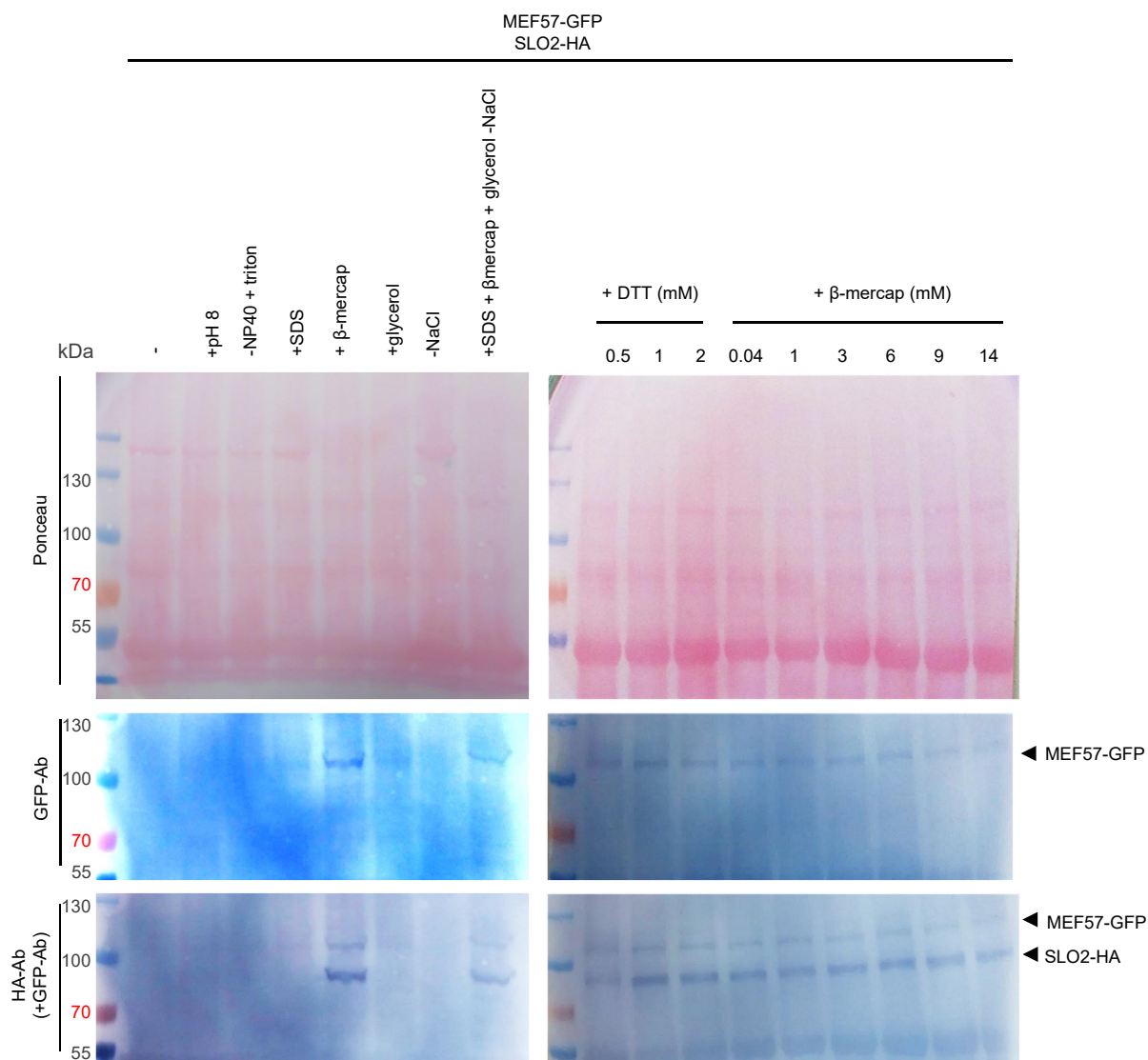

Supplement: S1 Fig — Protein ladder shown in the first run (170, 130, 100, 70 (red), 55, 40, 35, 25 and 15 kDa). The respective molecular weights were: SLO2-HA, 66.41 kDa; DYW2-GFP, 92.56 kDa; MEF57-GFP, 100.62 kDa; and HSP60.3B-GFP, 87.42 kDa. (PDF) [file pone.0187753.s001.pdf]
