# Supplementary material for: Optimization of non-denaturing protein extraction conditions for plant PPR proteins
Source: PLoS One. 2017 Nov 7;12(11):e0187753. doi: 10.1371/journal.pone.0187753 (PMC5675432; doi:10.1371/journal.pone.0187753)
Supplement: S2 Fig — Histograms showing the GFP/HA-tagged protein amount relative to Ponceau, according to band intensity in the blots. (PDF) [file pone.0187753.s002.pdf]

S2 Fig. GFP/HA-tagged protein amount relative to Ponceau. Histograms showing the GFP/HA-tagged protein amount relative to Ponceau, according to band intensity in the blots.

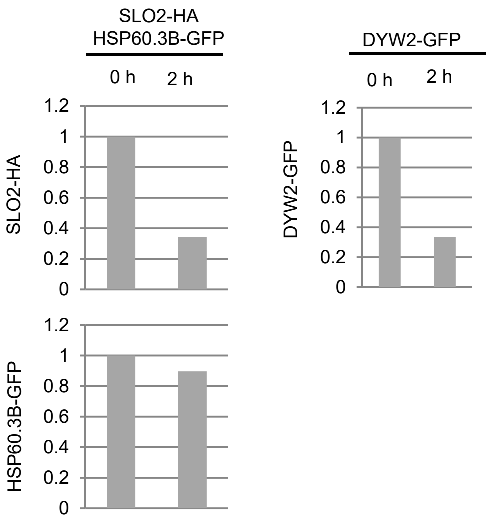

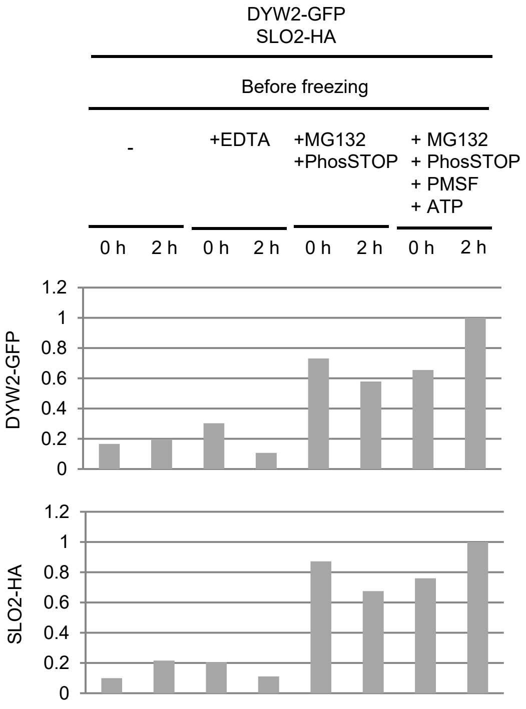

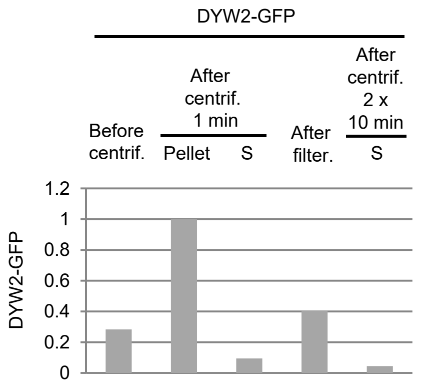
DYW2-GFP
Before centrif.
After centrif. 1 min
Pellet
S
After filter.
After centrif. 2 x 10 min
S
DYW2-GFP
1.2
1
0.8
0.6
0.4
0.2
0

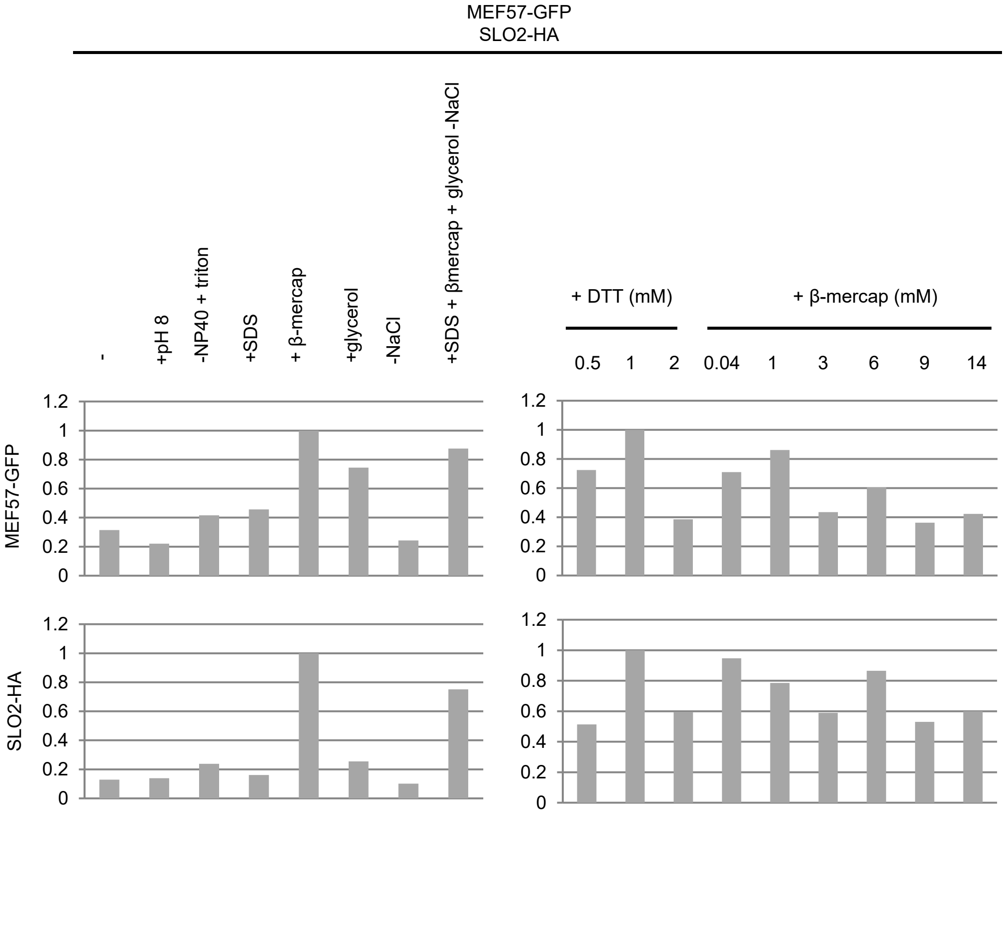
MEF57-GFP
SLO2-HA
-
+pH 8
-NP40 + triton
+SDS
+ β-mercap
+glycerol
-NaCl
+SDS + βmercap + glycerol -NaCl
+ DTT (mM)
+ β-mercap (mM)
0.5
1
2
0.04
1
3
6
9
14
MEF57-GFP
SLO2-HA
1.2
1
0.8
0.6
0.4
0.2
0
