# Supplementary material for: Optimization of non-denaturing protein extraction conditions for plant PPR proteins
Source: PLoS One. 2017 Nov 7;12(11):e0187753. doi: 10.1371/journal.pone.0187753 (PMC5675432; doi:10.1371/journal.pone.0187753)
Supplement: S1 Table — (PDF) [file pone.0187753.s003.pdf]

S1 Table. Oligonucleotides used for cloning.

Locus	gDNA/cDNA (bp)	Primer	Sequence (5` - 3`)
**At2g13600 (SLO2)**	2094/2094	attb-SLO2-F	GGGGACAAGTTTGTACAAAAAAGCAGGCTTCATGGCAACAAAATCATTTCTC
		attb-SLO2	GGG GAC CAC TTT GTA CAA GAA AGC TGG GTC CAT GGC GTT GTC CCA AAG AAG
**At2g15690 (DYW2)**	1740/1740	attb575-F	GGGG ACA AGT TTG TAC AAA AAA GCA GGC TTCATGTCTTCTCTAATGGCCATTCGTT
		attb575-R	GGG GAC CAC TTT GTA CAA GAA AGC TGG GTC CCAGTAATCCCCGCAAGAACAT
**At5g44230 (MEF57)**	1974/1974	attb547-F	GGGGACAAGTTTGTACAAAAAAGCAGGCTTC ATGACGGTGGCTCATTCTC
		attb547-R	GGG GAC CAC TTT GTA CAA GAA AGC TGG GTC CCAAAAATCACCACAAGAACAATC
**At3g23990 (HSP60.3B)**	3266/1734	attB-F240F	GGGGACAAGTTTGTACAAAAAAGCAGGCTTCATGTATCGTTTCGCTTCTAACC
		attB-R240R	GGGGACCACTTTGTACAAGAAAGCTGGGTCGTAGTCCATGCCTCCCATTCCACC
